# Platelet-Rich Plasma for Rheumatoid Arthritis: A Case Series

**DOI:** 10.7759/cureus.19629

**Published:** 2021-11-16

**Authors:** Dana Shively, Neel Amin

**Affiliations:** 1 Medicine, Florida International University Herbert Wertheim College of Medicine, Miami, USA; 2 Anesthesiology and Pain Management, University of Washington, Seattle, USA

**Keywords:** platelet-rich plasma (prp), rheumatoid arthitis, novel therapy, interphalangeal joint, chronic pain management

## Abstract

Rheumatoid Arthritis (RA) is a chronic disease characterized by severe inflammation that leads to degradation of articular cartilage and the formation of bony erosions. Currently, certain anesthesiologist-led pain management clinics have begun to take on a collaborative role in the treatment of patients with RA, as this progressive disease impairs work capacity due to chronic pain. We present three clinical cases in which platelet-rich plasma (PRP) was used for the treatment of RA in patients seeking a new therapy for pain control and improved range of motion, specifically in certain joints of the hand. The Patient Activity Scale II was employed as a standardized method to assess RA disease severity, recorded on the day of injection, at one month, at three months, and at six months. All of the included patients, ages 49, 60, and 63, had an established diagnosis of RA affecting the proximal interphalangeal and metacarpophalangeal joints of the hand. Over the course of six months, two out of three patients reported a 20% reduction in pain from the initial visit and a 30% improvement in overall well-being. The third patient noted a 50% decrease in pain from the initial visit and a 50% improvement in overall well-being. PRP treatment consistently resulted in functional improvement for each of the three patients treated, while also reducing long term pain and inflammation. Initial clinical and laboratory studies have shown that autologous plasma rich in platelets serves as a source of an abundance of growth factors once activated. The multitude of these growth factors injected into and around the diseased joints improves functionality in patients with RA indicating PRP may be a safe and beneficial therapy in patients with RA primarily affecting the joints of the hand.

## Introduction

Rheumatoid Arthritis (RA) is a chronic disease characterized by severe inflammation that leads to degradation of articular cartilage and the formation of bony erosions. A patient with RA primarily presents with significant swelling in the wrists, metacarpophalangeal, metatarsophalangeal, and proximal interphalangeal joints. Unfortunately, this condition affects about five in 1000 individuals worldwide, with a peak incidence in the sixth decade of life. Notably, women are two to three times more likely than men to develop RA over the course of their lifetime [1]. Despite continued advancements in treatment modalities, patients with RA continue to report a significantly diminished quality of life, as the condition interferes with daily physical functioning and productivity.

Therapeutic approaches today primarily consist of disease modifying antirheumatic drugs (DMARDs). These agents have been noted to be key components in managing a patient’s RA immediately after the initial diagnosis [1]. However, the most prescribed DMARDs are often not suitable for long-term management due to lack of lasting efficacy, toxicity, expense, and lack of insurance coverage, all of which contribute to discontinuation by patients [2-4]. Additionally, multiple adjuvant therapies targeted at symptomatic improvement for flare-ups such as glucocorticoids, nonsteroidal anti-inflammatory drugs (NSAIDs), and analgesics provide limited improvement in the progression of erosion and irreversible joint damage [1]. Together, these findings have stimulated research into more targeted and effective therapies for RA that produce more lasting, successful results.

Today, anesthesiologist-led pain management clinics have started to take a more active role in the management of patients with chronic pain as this progressive disease impairs work capacity, leads to disability, and impacts a patient’s psychological state due to chronic pain [2,5]. The utilization of platelet-rich plasma (PRP) as a regenerative medicine therapy in many common musculoskeletal pathologies has prompted consideration of its use in patients with RA. The effects of PRP have only been studied in limited trials for the treatment of anterior cruciate ligament tears, Achilles tendinopathy, epicondylitis, plantar fasciitis, cartilage regeneration, arthroplasty, bone healing, augmentation of spinal fusion, rotator cuff repair and osteoarthritis. Additionally, there is even more limited knowledge regarding the efficacy of PRP therapy in patients with RA and its use has not been studied extensively [6-9]. It has been hypothesized that due to the high concentration of platelets and growth factors, injection of this autologous blood sample may help diminish inflammatory factors, as well as accelerate neovascularization in joints with significant damage from this progressive disease [10]. Specifically, PRP could potentially improve joint homeostasis by limiting hyperplasia within the synovial membrane and decreasing cytokine levels without disrupting the innate cartilage tissue resulting in clinical improvement [6,11].

We present three clinical cases in which PRP was used for the treatment of RA in patients seeking a new therapy to control their chronic disease. Moving forward we believe that PRP therapy can serve as an additional treatment option, in which the body can use inherent physiologic tools to heal itself after the direct infusion of platelet-rich plasma.

## Case presentation

Methods

Three patients presenting with RA were treated with activated PRP. At the bedside, peripheral venous whole blood was drawn into 12 mL tube and PRP was created using a Juventix centrifugation systems (Juventix Regenerative Medical, Tampa, Florida) protocol by spinning at 3500 revolutions per min for eight minutes, to concentrate the platelets within the tube. Processing allows the visible gel separator within the tube to isolate the platelet-rich plasma clearly above the red blood cells and granulocytes for easy extraction. Next, a 32-gauge meso needle is placed at the top of the vial. An 18-gauge draw needle is then attached to a 10 mL syringe to withdraw the sample, directing the needle directly above the gel separator, thus extracting the entire platelet component.

Patients were positioned in a manner that made their injection sites readily accessible for the administration of serum. For injection into the joint, a 7 mL sample of PRP was mixed with 1 mL of lidocaine into a syringe and attached to a 25-gauge needle. Each patient received a standard 0.5 mL dose intra-articularly and a 1.5 mL dose peri-articularly in each affected joint.

In addition to clinical examination, the Patient Activity Scale II (PAS-II) was employed as a standardized method to assess RA disease severity. The PAS-II has been studied to be an effective and appropriate generic self-report indicator to assess active rheumatic disease [12].

The survey was recorded on the day of PRP injection, at one month, at three months, and lastly, at six months. The following two questions were primarily used to determine disease severity throughout the course of this report: “How much pain have you had because of your illness in the past week on a scale of 0 to 10?” and “Considering all the ways that your illness affects you, rate how you are doing on the following scale of 0 to 10.” The goal of each of these questions was to serve as an effective assessment tool based on the patient’s mobility and functional capacity to quantify disease activity using patient reported outcomes. The numerical scores were evaluated and compared at each of the four time points.

Results

Case 1

A 49-year-old male diagnosed with RA in 2019 presented to the clinic after previously declining initiation of DMARD therapy to treat his condition and subsequently struggled with significantly decreased range of motion, primarily in his hands, for one year before seeking pain management therapy. The patient came to the office in November of 2020 seeking therapeutic treatment of persistent inflammatory RA of the right fourth and fifth metacarpophalangeal joints. At his initial visit, the patient was given a PAS-II survey to complete. He noted “some difficulty” upon standing from a chair, doing outside work, and moving/lifting heavy objects, and reported he could complete the remaining activities “with no difficulty” such as opening car doors and reaching for objects. He reported 7/10 pain with his illness in the previous week. Additionally, he reported feeling at a level of 6/10 when asked about the effect of his illness on his daily functional capacity. On physical exam, the aforementioned joints were visibly swollen and tender to palpation. Per our methods, the patient received 0.5 mL intra-articularly and 1.5 mL peri-articularly.

After one month, the patient was re-evaluated and objectively reported increased range of motion and diminished pain in the areas treated with PRP therapy. At three months, using the PAS-II scale, the patient reported only 3/10 pain with his illness and 4/10 overall, in terms of his illness. By six months, the patient felt as though the procedure gave him 100% relief and he rated on PAS-II scale being at 2/10 for pain and at a 1/10 as a whole, in light of the illness. Overall, the patient reported a 50% decrease in pain compared to his initial visit and overall well-being regarding the scope of his RA.

Case 2

A 60-year-old female diagnosed with RA in 2018, who had declined DMARD therapy with her rheumatologist, was seen in the pain management office in November of 2020 for treatment of her continued pain and inflammation primarily affecting the right third proximal interphalangeal (PIP) joint (Figure 1). At the initial visit, the patient was given a PAS-II survey to complete. She noted “much difficulty” with grasping and moving heavy objects. She reported 6/10 pain from her RA within the past week and again a 6/10 overall rating for her illness. Per our methods, the patient received 0.5 mL intra-articularly and 1.5 mL peri-articularly.

After one month, the patient was evaluated again and objectively reported a mild decrease in her pain level, though still with significant restriction in her full range of motion. At three months, using the PAS-II scale, the patient reported 5/10 pain level and 5/10 overall, in terms of her illness. By six months, her PAS-II scale ratings were a 4/10 in terms of pain, and a 3/10 rating for overall daily functional capacity with her disease. Overall, she reported a 20% decrease in pain compared to her initial visit and 30% improvement in overall well-being in the scope of her RA.

Case 3

A 63-year-old male was diagnosed with RA in 2020 and was referred from his primary care physician to a rheumatologist for management of his condition. He was initially prescribed meloxicam for pain relief and then further sought therapy at our pain management clinic. He was seen in the office in November of 2020 for PRP administration in his afflicted joints - right third metacarpophalangeal (MCP) and right fifth PIP joints. At the initial visit, the patient was given a PAS-II survey to complete. He noted “some difficulty” with opening car doors and doing outside work such as yard work. He reported 4/10 pain from her RA within the past week and again a 4/10 overall illness rating. Per our methods, the patient received 0.5 mL intra-articularly and 1.5 mL peri-articularly into the affected joints.

After one month, the patient was re-evaluated and objectively noted an improvement in his range of motion, with only a minor reduction of pain. At three months, using the PAS-II scale, the patient reported 3/10 pain level and 3/10 overall illness rating. By six months, his PAS-II scale ratings were a 2/10 in terms of his pain and a 1/10 regarding the effect the disease had on his overall well-being. Overall the patient reported a 20% decrease in pain compared to his initial visit and 30% improvement in overall daily, functional capacity in terms of his RA.

## Discussion

Over the past decade, the application of PRP has substantially grown as a biological treatment for healing and regeneration of damaged tissues. Its effective pathophysiologic response has the potential to mitigate the significant damage caused by RA. Activation of the fibroblast-like synoviocytes (FLS) leads to the formation of “pannus,” a hyperplastic synovial lining that invades the periarticular bone leading to erosions and cartilage degradation [1]. Additionally, pro-inflammatory cytokines, such as tumor necrosis factor (TNF) and interleukin (IL)-6 mediate the symptoms of the disease including pain and inflammation. These molecules, which are inhibited by PRP, also generate osteoclasts within the synovial membrane, promoting further bone damage that manifests as significant joint swelling [13].

Current treatment options are aimed at the reduction of pain and inflammation to improve the quality of life of these patients, as this disease significantly affects activities of daily living. Since early diagnosis and treatment initiation halts the progression of joint damage in 90% of patients with early RA, a timely diagnosis and effective intervention is critical [1]. PRP has the potential to serve as a first-line treatment for these patients as it can regulate the migration, invasion, and adhesion of the characteristic FLS on the extracellular matrix by influencing the reorganization of the actin cytoskeleton and increasing matrix metalloproteinase-1 [14]. PRP itself is a volume of plasma fraction from autologous blood that contains a platelet concentration above baseline, ranging from 300,000 to 1,000,000 platelets/mL [15, 16]. American Association of Blood Banks has defined PRP as the plasma fraction resultant of a single light spin of whole blood, which is richer in platelets in comparison with other cell types. At baseline levels, platelets function as a reservoir for numerous types of growth factors including transforming growth factor-beta 1 (TGF-β1), vascular endothelial growth factor (VEGF), basic fibroblast growth factor (FGF), platelet-derived growth factor (PDGF), connective tissue growth factor (CTGF) and epidermal growth factor (EGF), interleukin 1b and IL-6 [9, 16]. When concentrated and directly injected, they have remarkable clinical potential for wound healing and tissue regeneration.

The benefits of PRP in the pathogenesis of repair and regeneration in the setting of RA has been largely evaluated in laboratory, preclinical studies. Tong et. al. administered PRP in human rheumatoid FLS cells and found that it effectively altered the production of inflammatory mediators and enhanced neovascularization, which likely accelerates the healing process. PRP also regulates key factors in the P13K/AKT pathway, which is significantly involved in the progressing pathogenesis of the disease [10]. Most notably, the migration of RA-FLS into joint spaces has been widely recognized as the pivotal step in the toxic nature of RA, thus by regulating the invasion of these cells, PRP therapy has the potential to alter deleterious progression of the disease. Platelet-rich plasma serves as a novel therapeutic treatment directly aimed at minimizing the destructive progression of RA. This conclusion is further supported by the array of randomized controlled clinical trials that have all demonstrated statistically significant improvement in patients’ clinical functionality after receiving PRP injections for various musculoskeletal pathologies. The conclusions drawn from these trials may likely be due to the observed, beneficial effects of PRP in the pathogenesis of healing [17-21].

Much of the clinical data supporting the use of therapeutic PRP has attempted to shed light on the benefits of PRP in treating patients with Osteoarthritis (OA), which shares some similarities in pathogenesis compared to RA, in regards to the pathophysiology of cartilage matrix degradation and progressive joint remodeling [8, 22]. Key cytokines, chemokines, proteases, cell adhesion molecules, and angiogenic factors are common in each pathological process. A 2013 quantitative synthesis of data from randomized control trials showed that patients treated with sequential intra-articular PRP injections had significant improvement at six months compared to those who received hyaluronic acid or normal saline using the Western Ontario and McMaster Universities Arthritis Index scale (mean difference, -18.0 [95% confidence interval, -28.8 to -8.3]; P < .001) [23]. A smaller study in 2017, also published similar results in 24 elderly patients with minor to moderate knee OA combined with supra-patellar bursitis. After just the second PRP injection, synovial fluid analysis revealed a two-fold decrease in inflammatory proteins such as matrix metalloproteinase, apolipoprotein A-I, and transferrin [24]. Both studies further support the idea that PRP injections can play a major role in combating degenerative joint processes inflicted by certain diseases.

This report focuses on the use of PRP in RA patients seeking treatment to improve joint pain and inflammation. We present these cases to support the idea that PRP should be considered as an additional therapeutic option in the treatment of both the major biochemical and clinical effects of RA. In our experience, 0.5 mL of intra-articular PRP and 1.5 mL of peri-articular PRP resulted in significant functional improvement six months after injection with promise of sustained improvement. These results were independent of the duration of disease and past or ongoing treatment for these patients. Additionally, no adverse reactions were noted over the six-month follow-up period in the three patients who received PRP treatment. There are very few serious adverse events reported for PRP in the literature, likely due to the autologous nature of the treatment [25,26]. Limitations of this study inherently arise from the small sample size of this case series. Additionally, the lack of access to past specific rheumatologic data for each patient, such as labs and imaging, limited our ability to objectively quantify the severity of initial disease, as well as the evaluation of objective measures of healing. Future research should be aimed at conducting well-planned randomized control trials evaluating the clinical safety and efficacy of PRP as a first-line treatment for RA. Further studies should also be directed at analyzing the optimal volume of PRP and platelet concentration necessary for maximal clinical benefits, as well as the timing of treatment.

## Conclusions

In conclusion, the precise mechanism of action of platelet-rich plasma shows promise as an effective agent for the management of RA in patients seeking an adjunct or alternative treatment to DMARD therapy. Initial clinical and laboratory studies have shown that autologous plasma rich in platelets serves as a source of an abundance of growth factors once activated. The multitude of these growth factors directly injected into the diseased joint accelerates healing and improves functionality in patients with RA. PRP is likely to be a safe and beneficial therapy in patients with any stage of RA. Intra-articular and peri-articular administration of PRP in outpatient settings is an easily adoptable treatment strategy, with growing emergence in our Board Certified Pain-Medicine practices. Overall, we believe that PRP should be further evaluated in clinical trials to outline the long-term clinical benefits in the improvement of joint structure and composition, as well as to evaluate the significant improvement expected in quality of life in patients with RA.
